# Efficacy of combined treatment with pasireotide, pegvisomant and cabergoline in an acromegalic patient resistant to other treatments: a case report

**DOI:** 10.1186/s12902-018-0231-9

**Published:** 2018-01-24

**Authors:** A. Ciresi, S. Radellini, V. Guarnotta, C. Giordano

**Affiliations:** Section of Endocrinology, Diabetology and Metabolic Diseases, Biomedical Department of Internal and Specialist Medicine (DIBIMIS), University of Palermo, Piazza delle Cliniche 2, 90127 Palermo, Italy

**Keywords:** Pasireotide, Pegvisomant, Acromegaly, Cotreatment, Resistant

## Abstract

**Background:**

The approach to acromegalic patients with persistent acromegaly after surgery and inadequate response to first-generation somatostatin receptor ligands (SRLs) should be strictly tailored. Current options include new pituitary surgery and/or radiosurgery, or alternative medical treatment with SRLs high dose regimens, pegvisomant (PEG) as monotherapy, or combined therapy with the addition of PEG or cabergoline to SRLs. A new pharmacological approach includes pasireotide, a second-generation SRL approved for patients who do not adequately respond to surgery and/or for whom surgery is not an option. No reports on efficacy and safety of combined therapy with pasireotide and pegvisomant (PEG) in acromegaly are available.

**Case presentation:**

Here we report the case of a 41-year-old acromegalic man with a mixed GH/PRL pituitary adenoma post-surgical resistant to first-generation SRLs both alone and in combination with cabergoline and PEG who achieved biochemical and tumor control with the combined triple treatment with pasireotide, PEG and cabergoline without adverse events and with a good compliance to treatment.

**Conclusions:**

Twelve months of therapy with pasireotide, PEG and cabergoline proved to be safe and effective in this particular patient and the clinical improvement of disease resulted in an improved compliance to treatment.

## Background

In acromegaly, medical therapy is recommended in patients with persistent or recurrent disease following surgery. The medical treatment includes long-acting somatostatin receptor ligands (SRLs), dopamine agonists (usually cabergoline) and the GH receptor antagonist [pegvisomant (PEG)] [1]. The first-generation SRLs have high affinity for somatostatin receptors (SSTR)2 and weak to moderate affinity for SSTR3 and SSTR5 [2].

The percentage of achievement of biochemical control widely varies in the different studies. Actually, without the use of stringent inclusion criteria required for clinical trials, in unselected treatment-naïve acromegalic patients a biochemical control can be achieved in a percentage which is far lower than those reported in the past [3], while real life studies indicate a biochemical control rate around 40% [4].

Resistance to SRLs may be defined as a failure to achieve biochemical control criteria (GH < 1.0 μg/L and a normal age-adjusted IGF-1) and tumor volume increase or absence of > 20% decrease after at least 12 months of treatment [5].

The approach to patients with persistent acromegaly after surgery and inadequate response to first-generation SRLs should be strictly tailored [6]. Current options include new pituitary surgery (in a patient with persistent disease and residual intrasellar adenoma following initial surgery), use of either a SRLs or PEG (as the initial adjuvant medical therapy in a patient with significant disease without local mass effects), a trial of a dopamine agonist, usually cabergoline (as the initial adjuvant medical therapy in a patient with modest elevations of serum IGF-1 and mild signs and symptoms of GH excess), the combined therapy with the addition of PEG or cabergoline (in a patient with inadequate response to SRLs) or, finally, the use of radiation therapy (in the setting of residual tumor mass following surgery if medical therapy is unavailable, unsuccessful or not tolerated) [1].

The switch to treatment with PEG as monotherapy or in association with SRLs represents nowadays a valid therapeutic strategy to achieve full disease control in acromegalic patients resistant or poorly responders to SRLs [1, 7].

PEG has been shown to control acromegaly in 60–90% of patients across several clinical trials. If in the real life the rate of disease control was lower, not exceeding 65–70%, an appropriate PEG dose titration up to the maximum allowed dosage was shown to normalize IGF-1 levels in up to 90% of cases even in the real life setting [3, 8, 9].

A new pharmacological approach includes pasireotide, a second-generation SRL approved for patients who do not adequately respond to surgery and/or for whom surgery is not an option [10].

Unlike first-generation SRLs which primarily exert their effects through binding to SSTR2, pasireotide binds with high affinity to SSTR5 [11].

Pasireotide has proven to be superior to first-generation SRLs both as first line medical treatment in naïve patients [12] and in patients classified as resistant [13].

To our knowledge, although the outcomes of combined therapy with the addition of PEG to first-generation SRLs are widely known, there are no reports available on the efficacy and safety of combined therapy with pasireotide and PEG. Here we report our experience with this new combined treatment in an acromegalic patient.

This study was carried out in accordance with the recommendations of Endocrine Society Clinical Practice Guidelines and at the time of hospitalization a written informed consent was obtained from the patient for publication of this case report and any accompanying images, in accordance with the Declaration of Helsinki.

## Case presentation

A 41-year-old man with clinically evident acromegaly was referred to our department on his own initiative due to the physical changes over the years that had been noticed by the new wife by looking at past pictures. He stated that 1 year before had undergone surgery for carpal tunnel syndrome and for some months he reported snoring, profuse sweating, joint aches and occasional headache. He denied to have hypertension, galactorrhea, signs and symptoms of diabetes mellitus.

At clinical examination, the patient showed a clear acromegalic phenotype, with prominence of the brow, enlargement of the nose, thickening of the lips, prognathism, macroglossia, increased interdental spacing, acral enlargement, evident thyroid goiter. He showed blood pressure values into normal range, normal cardiac and respiratory exam, a mild splenomegaly.

Initial testing revealed IGF-1 1369 μg/l (normal: 109-204), basal-GH 31 μg/l with GH-nadir during an oral glucose tolerance test (OGTT) 16 μg/l, prolactin 2386 ng/ml (normal: < 15.2 ng/ml), total testosterone 0.88 μg/l (normal: 2.4-9.3 μg/l) with normal gonadotropins, parathyroid hormone 52 pg/ml (normal: 15-65 pg/ml), normal adrenal and thyroid function. The patient showed normal glucose tolerance (fasting glucose 4.78 mmol/l), with mild hyperinsulinism (OGTT-peak 230.6 μU/ml), glycosylate hemoglobin (HbA1c) 5.9%) and normal lipid profile. Magnetic resonance imaging (MRI) revealed a large hyperintense mass of 28,512 mm^3^ (largest diameter 33 mm) with supra- and latero-sellar extension (with bilateral, mostly left, involvement of the cavernous sinus). Campimetry showed monolateral inferior quadrantanopsia.

The patient refused surgery as a first line treatment and started treatment with cabergoline (0.5-1.0 mg/weekly) and lanreotide-autogel 90 mg/monthly.

After 3 and 6 months he showed, respectively, IGF-1 1049-908 μg/l, basal-GH 35.6-44 μg/l; prolactin 1526-959 ng/ml. No side effects were reported, except for a quite significant increase in fasting glucose (6.44 mmol/l) and HbA1c (6.5%). At 6 months MRI revealed a volumetric tumor reduction (23,500 mm^3^) with depression of the superior profile. Treatment was modified by increasing cabergoline to 1.5 mg/weekly and lanreotide to 120 mg/monthly for 6 months. A further, very small, decrease in hormonal levels was obtained, but without reaching the target, as follows: IGF-1850 μg/l, basal-GH 32 μg/l, prolactin 589 ng/ml. MRI showed an almost unchanged tumor mass. Fasting glucose (6.22 mmol/l) and HbA1c (6.5%) did not significantly change.

The patient was persuaded to undergo transsphenoidal adenomectomy, without complications, in july 2012. Histologically, the tumor was classified as GH/PRL and sparsely-granulated type, with a Ki-67 labeling index < 1%.

Despite a significant debulking of tumor, surgery was not curative but it probably led to a better biochemical response to subsequent medical treatment. The early random-GH after 1 month was 47 μg/L and the evaluation at 3 months confirmed persistent disease, with IGF-1631 μg/l, random-GH 15.2 μg/L, nadir-GH 11.5 μg/L. PRL was 251.9 ng/ml, total testosterone slightly increased (1.85 μg/l) and glucose metabolism back in the norm (fasting glucose 5.17 mmol/l, HbA1c 6.0%, insulin OGTT-peak 132.6 μU/ml). MRI revealed a residual tumor with left extension and campimetry was normal.

The patient started adjuvant therapy with octreotide-LAR and cabergoline, with the dosage progressively increased (maximum doses octreotide 40 mg/monthly and cabergoline 2.25 mg/weekly). This treatment did not result in hormonal normalization, as shown by random-GH 10.9 μg/L, IGF-1549 μg/l, PRL 76 ng/ml.

In august 2013 PEG was added to octreotide with a 3-times-a-week schedule as follows: 60 mg/weekly for 4 months, 90 mg/weekly for other 4 months and 120 mg/weekly for just 3 months (cabergoline dose unchanged). A dose-responsive IGF-1 reduction (437, 410 and 305 μg/l, respectively) was observed as the PEG dose gradually increased, although IGF-1 and PRL (72 ng/ml) remained above the norm. No significant change in tumor volume was seen and the glucose metabolism slightly improved (last HbA1c 5.8%). However, PEG was stopped by the patient due to lack of clinical improvement and poor compliance of patient to PEG treatment in the following weeks.

In july 2014, as there was still no commercial use of pasireotide, we had the opportunity to use it as compassionate treatment. We replaced octreotide with pasireotide-LAR 40 mg/monthly (cabergoline dose unchanged). After 6 months random-GH was 12.07 μg/L, IGF-1613 μg/L and PRL 41.2 ng/ml, with increased fasting glucose (6.17 mmol/l) and HbA1c (6.4%). MRI showed a decrease in tumor volume (15,525 mm^3^) and a heterogeneous signal intensity, with predominant hyperintensity, on both T1 and T2-weighted images compatible with presence of necrotic areas.

Pasireotide was increased to 60 mg/monthly for 4 months without significant biochemical benefits: random-GH 10.9 μg/L, IGF-1518.9 μg/L.

In june 2015, after obtaining informed consent from the patient who refused surgery, PEG was added to pasireotide with a 6-times-a-week schedule, with a starting daily dose of 10 mg (60 mg/weekly), gradually increased to 15 (90 mg/weekly) up to 20 mg (120 mg/weekly) after 3 and 6 months (cabergoline dose unchanged). After 3 months of PEG-10 mg, 3 months of PEG-15 mg and 6 months of PEG-20 mg, IGF-1 was 344-234-172 μg/L and PRL 30-28.5-19 ng/ml, respectively. Fasting glucose was 6.5-5.06-5.39 mmol/l, while HbA1c changed from 6.4 to 6.8-5.9-5.5%. After 12 months, MRI revealed an unchanged tumor volume. Clinically, an improvement in tiredness and joint pain was reported. The patient’s compliance was satisfactory throughout the whole period of treatment and no side effects were reported. To date, the patient is continuing the treatment with the latest available IGF-1174 μg/L and PRL 9.5 ng/ml.

**Fig. 1:**
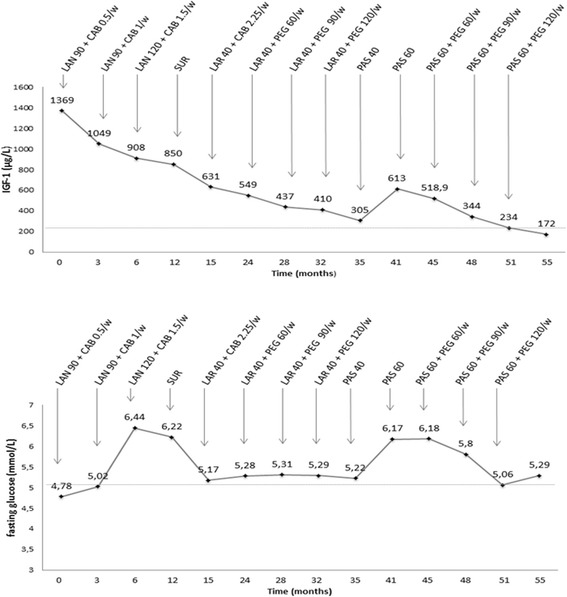
IGF-1 (top) and fasting glucose (bottom) levels during the entire clinical follow-up of the patient. *LAN: lanreotide autogel; CAB: cabergoline; SUR: surgery (transsphenoidal adenomectomy); LAR: octreotide LAR; PEG: pegvisomant; PAS: pasireotide; w: weekly. All drugs doses are expressed in mg*

IGF-1 and fasting glucose levels during the entire clinical follow-up of the patient are presented in the Fig. 1.

**Fig. 2:**
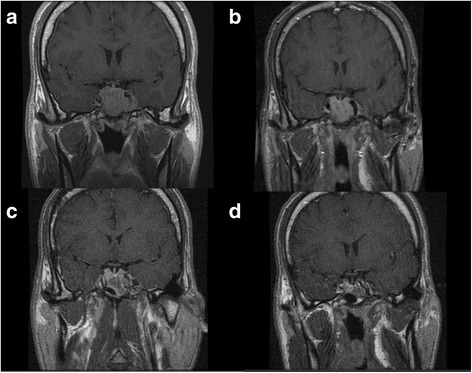
Sellar MR coronal images at diagnosis (**a**), after 6 months of lanreotide autogel (**b**), 3 months after surgery (**c**) and 6 months after pasireotide treatment (**d**)

MR coronal images at diagnosis and during the follow-up of the patient are presented in the Fig. 2.

## Discussion

We describe for the first time the effectiveness of combined treatment with pasireotide and PEG in a patient with post-surgical active disease resistant to combined treatment with first-generation SRLs and PEG.

It is widely accepted that the post-surgical management of acromegalic patients with residual disease should be tailored. In patients with active disease the use of SRL as initial adjuvant medical therapy is suggested, with the subsequent addition of PEG or cabergoline (this latter in case of mild disease activity) to SRL in patients with inadequate response to an SRL [1].

According to these guidelines, following surgery we first started octreotide LAR up to the maximum monthly dosage. The poor response to octreotide as an adjuvant therapy was probably expected because of the biochemical resistance already shown to lanreotide before surgery. Indeed, octreotide and lanreotide seem to be equally effective in the biochemical control of acromegaly [14].

However, surgical debulking may significantly improve subsequent response to medical therapy, particularly in cases of highly active disease [15] as in this case, but when at least 75% of the tumor is removed [16], while here the debulking was much lower.

This patient had many determinants of a poor response, such as age, tumor volume, high baseline hormonal levels and sparsely granulated adenoma [17] and these parameters probably should have led us to anticipate other therapeutic decisions.

In addition, low tissue SSTR2 expression as a determinant of poor response to SRLs can not be excluded. Indeed, the expression of an adequate amount of SSTR2 is a requisite for response to treatment [18]. Unfortunately the characterization of SSTR subtypes was not performed in this case. However, since pasireotide monotherapy was not more effective than SRLs in decreasing IGF-1 levels, the better outcome can not be attributed only to this factor.

The switch to PEG as a monotherapy or in association with SRLs represents a valid strategy in resistant patients [1, 9]. Despite an initial optimal compliance to treatment, in our patient the addition of PEG to octreotide was able to reduce IGF-1 by just 20%, while the combined therapy with pasireotide resulted in a progressive decline in IGF-1 already after 3 months up to complete control, using the same weekly dose as already used in the first attempt at combination with octreotide.

Probably, both the different binding to SSTR [11] and the different behavior on post-SSTR intracellular cascade had a fundamental role. Indeed, pasireotide modulates SSTR trafficking differently than octreotide, resulting in quicker recycling of SSTRs, particularly SSTR2, and may counteract SSTR2-desensitization [10].

In addition, we could speculate that, despite the same weekly dose, the 6-times-a-week schedule of PEG administration, compared to the 3-times-a-week schedule, may have been more effective in IGF-1 reduction. The patient surprisingly showed better compliance probably because of the associated clinical improvement. Consequently, the better outcome can be attributed to a better dosing schedule and association with pasireotide rather than with a better compliance to pegvisomant treatment.

Of course, it is worth noting that this combined treatment, which must be practiced for the rest of life, is very expensive and the health economics issue and the cost-effectiveness of different treatments are important considerations in management decisions in acromegaly. For these reasons, in the near future we will again try to offer to the patient other therapeutic options, such as new surgery or radiotherapy, which at present the patient refused.

Similarly, we carefully informed the patient of the possible expected adverse events of current therapy, the main of which being the latent negative effect of pasireotide on glucose metabolism [19]. Indeed, the highest binding affinity of pasireotide for SSTR5 has an important role in mediating insulin secretion. Conversely, glucagon secretion is mainly mediated by SSTR2, and this may account for the more modest suppressive effect of pasireotide on glucagon secretion, which can lead to a deterioration of glucose metabolism. Although this effect to date seems to be well balanced by the favorable effect on glycemic profile of PEG, a close monitoring of glucose homeostasis will be necessary during the entire course of the follow-up.

## Conclusions

In conclusion, 12 months of therapy with pasireotide and PEG proved to be safe and effective in achieving biochemical and tumor control and clinical improvement of acromegaly resulted in improved compliance. This combined treatment may represent a valid therapeutic strategy to achieve full disease control in acromegalic patients resistant or poorly responders to first-generation SRLs. However, although in this particular patient the combined treatment was safe and effective, we are well aware that this is only one case and that we can not generalize safety issues based only in one patient. Clinical trials involving several patients and with longer periods of follow-up are needed to confirm these findings.

## Abbreviations

HbA1c: Glycosylate hemoglobin
MRI: Magnetic resonance imaging
OGTT: Oral glucose tolerance test
PEG: Pegvisomant
SRLs: Somatostatin receptor ligands
SSTR: Somatostatin receptors
